# Activation of the trigeminal system as a likely target of SARS-CoV-2 may contribute to anosmia in COVID-19

**DOI:** 10.1177/03331024211036665

**Published:** 2021-08-18

**Authors:** Karl Messlinger, Winfried Neuhuber, Arne May

**Affiliations:** 1 Institute of Physiology and Pathophysiology, Friedrich-Alexander University, Erlangen-Nuernberg, Germany; 2 Institute of Anatomy and Cell Biology, Friedrich-Alexander University, Erlangen-Nuernberg, Germany; 3 Department of Systems Neuroscience, University Medical Center Eppendorf, Hamburg, Germany

**Keywords:** Coronavirus disease, headache, olfactory, trigeminal, anosmia

## Abstract

Clinical publications show consistently that headache is a common symptom in the coronavirus disease of 2019 (COVID-19). Several studies specifically investigated headache symptomatology and associated features in patients with COVID-19. The headache is frequently debilitating with manifold characters including migraine-like characteristics. Studies suggested that COVID-19 patients with headache vs. those without headache are more likely to have anosmia. We present a pathophysiological hypothesis which may explain this phenomenon, discuss current hypotheses about how the coronavirus SARS-CoV-2 enters the central nervous system and suggest that activation of the trigeminal nerve may contribute to both headache and anosmia in COVID-19.

Headache is a frequently described initial symptom of COVID-19 (1). The headache is mostly bilateral though it frequently has a migraine-like phenotype and lasts longer than two days in most cases (2,3). Remarkably, it shows a clear association with anosmia (4,5), however the pathophysiological basis for this association is as yet unclear. Meinhardt and colleagues (6) presented postmortem findings in patients with COVID-19 and discussed how SARS-CoV-2 enters the central nervous system (CNS) and causes multiple neurological symptoms including headache and loss of smell and taste. They showed the presence of SARS-CoV-2 ribonucleic acid (RNA) and protein mainly in the nasal mucosa and certain brain areas. They suggested a transmucosal entry via regional nervous structures, possibly followed by transport along the olfactory tract of the CNS. The olfactory neuroepithelium shows a high expression of ACE2 receptors used by SARS-CoV-2 to enter the cells, which has been suggested to cause the early olfactory dysfunctions (7). Another nervous structure, the trigeminal nerve, has been suggested by Caronna et al. (5) and Bolay et al. (8) to be as likely to serve as a point of entry into the brain and if so, could not only explain the loss of taste and smell but also the headache associated with an infection of SARS-CoV-2 (Figure 1).

**Figure 1:**
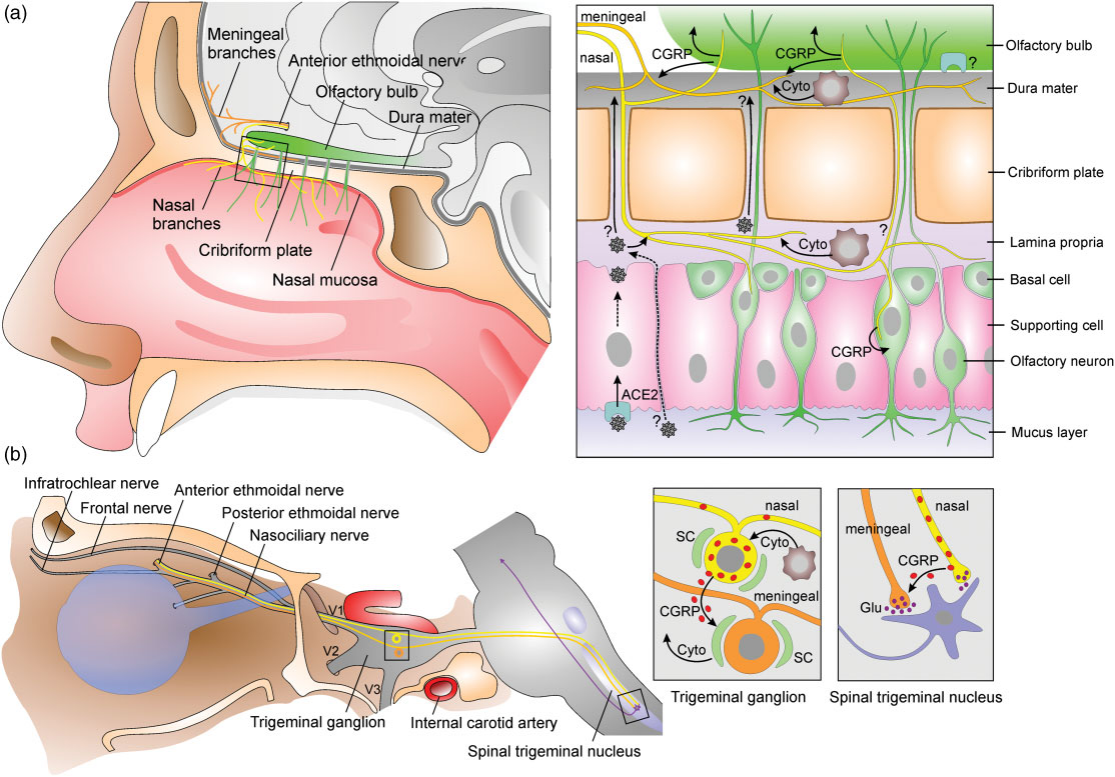
Schematic representation illustrating the hypotheses about the movement of SARS-CoV-2 and its effects on nasal olfactory and trigeminal nerve fibers (A) as well as the interaction of trigeminal afferents in the trigeminal ganglion and the spinal trigeminal nucleus (B) leading to headache and anosmia. SARS-CoV-2 binding to ACE2 receptors expressed by supporting cells in the nasal mucosa causes inflammatory and immune reactions involving the release of cytokines (Cyto) from various immune cells in the mucosa and likely also in the dura mater. Possible alternative ways of SARS-CoV-2 penetration into the CNS are the foramina of the cribriform plate or the infection of trigeminal afferents. Cytokines and inflammatory mediators sensitize and activate nasal and meningeal trigeminal afferents, which may also be cross-sensitized by CGRP. However, massive CGRP release from trigeminal afferents can also contribute to attenuating the olfactory function in the nasal mucosa and the olfactory bulb. Cross-sensitization of trigeminal afferents may in addition occur in the trigeminal ganglion, where CGRP can induce cytokine production in satellite glial cells (SC), and in the spinal trigeminal nucleus, where CGRP contributes to synaptic transmission, partly by enhancing glutamate (Glu) release from other central trigeminal terminals.

These hypotheses give rise to two principal questions. The first question is: how does the virus penetrate the brain and how it is transported? There are two possible routes for the direct passage of peptides from the nose to the brain, namely an intraneuronal pathway using the axonal transport, and an extraneural pathway through intercellular clefts in the olfactory epithelium directly connected with the subarachnoid space (9). Large molecules such as wheat germ agglutinin-horseradish peroxidase are known to hardly penetrate the blood-brain-barrier (BBB) but have been found in the CNS shortly after nasal application (10). Even large peptides such as melanocortin, vasopressin and insulin can be demonstrated in cerebrospinal fluid (CSF) within 30-80 minutes after intranasal application, probably bypassing the bloodstream (11). Triptans, which are the widely used treatment of choice for acute migraine and cluster headache attacks, do not or very sparsely penetrate the BBB, yet are effective through nasal application. More to the point, therapeutic concentrations in the CNS of a radiolabelled triptan (zolmitriptan), have been demonstrated shortly after nasal administration using positron emission tomography (PET) (12). An extracellular route via diffusion of SARS-CoV-2 particles through the cribriform plate between the nasal mucosa and the subarachnoidal space seems certainly possible and is supported by findings in mice showing that the CSF can leave the cranial cavity through the cribriform plate (13). On the other hand, there are several possibilities of neuronal transport once the virus gets into a neuron. At first glance, the fila olfactoria, the axons of the primary olfactory neurons, which enter the brain through the cribriform plate and synapse within the olfactory bulb, appear to be a target of such a transport. However, as mentioned above, the SARS-CoV-2 needs ACE2 receptors to enter the cells but these receptors seem not to be expressed by olfactory sensory neurons but rather by sustentacular cells and basal cells in rodent, primate and human nasopharyngeal mucosa (14,15), although some colocalization of SARS-CoV spike protein immunoreactivity with neural cells in olfactory mucosa samples has been found by Meinhardt et al. (6). Another receptor that binds SARS-CoV-2, the neuropilin-1 receptor (NRP1), which is expressed in olfactory neurons, has recently been suggested as an alternative way to enter the neurons (16). For the trigeminal afferents innervating the nasopharyngeal mucosa no data are currently available regarding ACE2 expression, although this appears possible; at least ACE2 expression has been found in a subset of human dorsal root ganglion neurons (17,18). According to the findings of Meinhardt et al. (6), the density of virus RNA in the olfactory bulb is comparable to the presence of RNA in the trigeminal ganglion, where the cell bodies of the trigeminal afferents are located. However, in the mouse ACE2 expression has been found in superficial layers of the olfactory bulb but not in any neuronal cells (15).

The second principal question is, why SARS-CoV-2 causes a loss of function (anosmia) in the olfactory system but a gain of function (headache) in the trigeminal system; and furthermore, are both these phenomena interconnected? Most of the trigeminal afferents innervating the nasal mucosa are nociceptors expressing transient receptor potential (TRP) cation channels of the type TRPV1, which may be activated by chemical compounds like capsaicin. Inflammatory responses caused by SARS-CoV-2 with a massive increase of cytokines in the nasal mucosa, particularly high TNF-α levels (19), may induce a cascade of nociceptive processes in the trigeminal system. TNF-α induces the expression of a variety of cytokines including IL-1β (20) as well as brain-derived neurotrophic factor (BDNF) (21) and TRPV1 receptor channels (22) in the trigeminal ganglion (TG). TNF-α and IL-1β stimulate expression and release of calcitonin gene-related peptide (CGRP) in TG neurons (23,24) and conversely, CGRP stimulates the expression of cytokines like IL-1β in TG satellite glial cells (25).

In rodents as well as humans, the afferent fibres from the nasal mucosa reach the anterior cranial fossa via the ethmoid nerve and travel in the dura mater to the trigeminal ganglion (26). Activation of these mucosal nerve endings following stimulation with capsaicin releases calcitonin gene-related peptide (CGRP) from the activated nociceptive afferents not only in the nasal mucosa but, importantly, also in the dura mater, leading to an increase in meningeal blood flow (27). If the mucosal trigeminal afferents passing through the dura mater are vigorously activated, the neuro-inflammatory cascades with increased neuropeptide release may spread to the dura mater involving the release of cytokines from macrophages (18) and secondarily activate meningeal nociceptors (28) to cause headache. Furthermore, the TG with multiple nociceptive interactions (29) and eventually the spinal trigeminal nucleus with converging afferent inputs from the nasal mucosa and the meninges (30) can be involved in the neuro-inflammatory cascades. Such a scenario may account for the acute headaches as well as the prolonged headaches accompanying the presumed cytokine storm in COVID-19 (5). The damage to the olfactory pathway caused by the virus or by the inflammatory process may cause anosmia on the one hand and stimulate the trigeminal system causing headache through the above interactions on the other hand. Lesions of the olfactory bulb following COVID-19 have been visualized by neuroimaging (31–33), substantiating structural changes that may underlie the anosmia. Inflammatory mediators produced and released through these lesions are likely to activate trigeminal afferents in the mucosa and the meninges surrounding the olfactory bulb.

However, the loss in olfactory function may not only be explained by the damage of the olfactory epithelium (34) but also by an involvement of the overactive trigeminal afferent system with an increased CGRP release, which in this context surprisingly has an inhibitory effect. The olfactory and trigeminal system are functionally connected and trigeminal activation is increased in patients with acquired anosmia (35). Part of this interaction may occur in the nasal mucosa, where it has experimentally been shown that CGRP released from activated trigeminal fibres inhibits the response of olfactory receptors to olfactory stimuli (36). In addition, from tracer experiments in rodents we know that trigeminal afferents innervating the nasal mucosa and travelling through the ethmoid nerve form collaterals innervating the olfactory bulb (37). There is experimental evidence that these trigeminal endings contribute to inhibitory effects on neurotransmission within the olfactory bulb, again by CGRP release (38). Thus, vigorous activation of trigeminal afferents injured by SARS-CoV-2 may contribute to both headache and concomitant anosmia.

In conclusion, the frequent association of headache and anosmia in COVID-19 may originate with inflammatory responses in the nasal mucosa but probably also requires the activation of meningeal nociceptors. SARS-CoV-2 may penetrate the cribriform plate to affect the meninges around the olfactory bulb. Although this is still a hypothesis that should be further investigated, CGRP released from activated trigeminal afferents may therefore contribute to suppress the olfactory functions in the nasal mucosa and the olfactory bulb in those patients experiencing simultaneously headache and anosmia

## References

[1] Bolay H Gül A Baykan B. COVID‐19 is a real headache! Headache 2020; 60: 1415–1421. 32412101 10.1111/head.13856 PMC7272895

[2] Sampaio Rocha-Filho PA Magalhães JE. Headache associated with COVID-19: Frequency, characteristics and association with anosmia and ageusia. Cephalalgia 2020; 40: 1443–1451. 33146035 10.1177/0333102420966770 PMC7645592

[3] Uygun Ö Ertaş M Ekizoğlu E , et al. Headache characteristics in COVID-19 pandemic-a survey study. J Headache Pain 2020; 21: 121. 33050880 10.1186/s10194-020-01188-1 PMC7552597

[4] Toptan T Aktan Ç Başarı A , et al. Case series of headache characteristics in COVID‐19: headache can be an isolated symptom. Headache 2020; 60: 1788–1792. 32790216 10.1111/head.13940 PMC7436308

[5] Caronna E Ballvé A Llauradó A , et al. Headache: A striking prodromal and persistent symptom, predictive of COVID-19 clinical evolution. Cephalalgia 2020; 40: 1410–1421. 33146036 10.1177/0333102420965157 PMC7645597

[6] Meinhardt J Radke J Dittmayer C , et al. Olfactory transmucosal SARS-CoV-2 invasion as a port of central nervous system entry in individuals with COVID-19. Nat Neurosci. *Epub ahead of print 30 November* 2020. DOI: 10.1038/s41593-020-00758-5. 10.1038/s41593-020-00758-5 33257876

[7] Chen M Shen W Rowan NR , et al. Elevated ACE-2 expression in the olfactory neuroepithelium: implications for anosmia and upper respiratory SARS-CoV-2 entry and replication. *Eur Respir J* 2020; 56: 2001948. 10.1183/13993003.01948-2020 PMC7439429 32817004

[8] Bolay H Özge A Uludüz D , et al. Are migraine patients at increased risk for symptomatic coronavirus disease 2019 due to shared comorbidities? Headache 2020; 60: 2508–2521. 33124044 10.1111/head.13998

[9] Illum L. Transport of drugs from the nasal cavity to the central nervous system. Eur J Pharm Sci 2000; 11: 1–18. 10913748 10.1016/s0928-0987(00)00087-7

[10] Thorne RG Emory CR Ala TA , et al. Quantitative analysis of the olfactory pathway for drug delivery to the brain. Brain Res 1995; 692: 278–282. 8548316 10.1016/0006-8993(95)00637-6

[11] Born J Lange T Kern W , et al. Sniffing neuropeptides: a transnasal approach to the human brain. Nat Neurosci 2002; 5: 514–516. 11992114 10.1038/nn849

[12] Wall A Kagedal M Bergström M , et al. Distribution of zolmitriptan into the CNS in healthy volunteers: a positron emission tomography study. Drugs R D 2005; 6: 139–147. 15869317 10.2165/00126839-200506030-00002

[13] Ma Q Ineichen BV Detmar M , et al. Outflow of cerebrospinal fluid is predominantly through lymphatic vessels and is reduced in aged mice. Nat Commun 2017; 8: 1434. 29127332 10.1038/s41467-017-01484-6 PMC5681558

[14] Bilinska K Jakubowska P Von Bartheld CS , et al. Expression of the SARS-CoV-2 entry proteins, ACE2 and TMPRSS2, in cells of the olfactory epithelium: identification of cell types and trends with age. ACS Chem Neurosci 2020; 11: 1555–1562. 32379417 10.1021/acschemneuro.0c00210 PMC7241737

[15] Brann DH Tsukahara T Weinreb C , et al. Non-neuronal expression of SARS-CoV-2 entry genes in the olfactory system suggests mechanisms underlying COVID-19-associated anosmia. *Sci Adv*. Epub ahead of print 31 July 2020. DOI: 10.1126/sciadv.abc5801. 10.1126/sciadv.abc5801 PMC10715684 32937591

[16] Hopkins C, Lechien JR, Saussez S. More that ACE2? NRP1 may play a central role in the underlying pathophysiological mechanism of olfactory dysfunction in COVID-19 and its association with enhanced survival. *Med Hypotheses* 2021; 146: 110406. 10.1016/j.mehy.2020.110406 PMC7678428 33246692

[17] Shiers S Ray PR Wangzhou A , et al. ACE2 and SCARF expression in human dorsal root ganglion nociceptors: implications for SARS-CoV-2 virus neurological effects. Pain 2020; 161: 2494–2501. 32826754 10.1097/j.pain.0000000000002051 PMC7572821

[18] McFarland AJ Yousuf MS Shiers S , et al. Neurobiology of SARS-CoV-2 interactions with the peripheral nervous system: implications for COVID-19 and pain. Pain Rep 2021; 6: e885. 33458558 10.1097/PR9.0000000000000885 PMC7803673

[19] Torabi A Mohammadbagheri E Akbari Dilmaghani N , et al. Proinflammatory cytokines in the olfactory mucosa result in COVID-19 induced anosmia. ACS Chem Neurosci 2020; 11: 1909–1913. 32525657 10.1021/acschemneuro.0c00249

[20] Durham ZL Hawkins JL Durham PL. Tumor necrosis factor-Alpha stimulates cytokine expression and transient sensitization of trigeminal nociceptive neurons. Arch Oral Biol 2017; 75: 100–106. 27836101 10.1016/j.archoralbio.2016.10.034 PMC5266621

[21] Bałkowiec-Iskra E Vermehren-Schmaedick A Balkowiec A. Tumor necrosis factor-α increases brain-derived neurotrophic factor expression in trigeminal ganglion neurons in an activity-dependent manner. Neuroscience 2011; 180: 322–333. 21335064 10.1016/j.neuroscience.2011.02.028 PMC3070813

[22] Khan AA Diogenes A Jeske NA , et al. Tumor necrosis factor α enhances the sensitivity of rat trigeminal neurons to capsaicin. Neuroscience 2008; 155: 503–509. 18582539 10.1016/j.neuroscience.2008.05.036

[23] Bowen EJ Schmidt TW Firm CS , et al. Tumor necrosis factor-alpha stimulation of calcitonin gene-related peptide expression and secretion from rat trigeminal ganglion neurons. J Neurochem 2006; 96: 65–77. 16277606 10.1111/j.1471-4159.2005.03524.x PMC1486866

[24] Lei L Yuan X Wang S , et al. Mitogen-activated protein kinase pathways are involved in the upregulation of calcitonin gene-related peptide of rat trigeminal ganglion after organ culture. J Mol Neurosci 2012; 48: 53–65. 22528462 10.1007/s12031-012-9772-y

[25] Afroz S Arakaki R Iwasa T , et al. CGRP induces differential regulation of cytokines from satellite glial cells in trigeminal ganglia and orofacial nociception. Int J Mol Sci 2019; 20: 711. 10.3390/ijms20030711 PMC6386987 30736422

[26] Finger TE Böttger B. Peripheral peptidergic fibers of the trigeminal nerve in the olfactory bulb of the rat. J Comp Neurol 1993; 334: 117–124. 7691899 10.1002/cne.903340110

[27] Gottselig R Messlinger K. Noxious chemical stimulation of rat facial mucosa increases intracranial blood flow through a trigemino-parasympathetic reflex–an experimental model for vascular dysfunctions in cluster headache. Cephalalgia 2004; 24: 206–214. 15009014 10.1111/j.1468-2982.2004.00649.x

[28] Zhang X-C Kainz V Burstein R , et al. Tumor necrosis factor-α induces sensitization of meningeal nociceptors mediated via local COX and p38 MAP kinase actions. Pain 2011; 152: 140–149. 21036476 10.1016/j.pain.2010.10.002 PMC3391748

[29] Messlinger K Balcziak LK Russo AF. Cross-talk signaling in the trigeminal ganglion: role of neuropeptides and other mediators. J Neural Transm 2020; 127: 431–444. 32088764 10.1007/s00702-020-02161-7 PMC7148261

[30] Koch M Sertel-Nakajima J Messlinger K. Responses of spinal trigeminal neurons to noxious stimulation of paranasal cavities – a rat model of rhinosinusitis headache. Cephalalgia 2021; 41: 535–545. 33203222 10.1177/0333102420970467

[31] Politi LS Salsano E Grimaldi M. Magnetic resonance imaging alteration of the brain in a patient with coronavirus disease 2019 (COVID-19) and anosmia. JAMA Neurol 2020; 77: 1028–1029. 32469400 10.1001/jamaneurol.2020.2125

[32] Laurendon T Radulesco T Mugnier J , et al. Bilateral transient olfactory bulb edema during COVID-19-related anosmia. Neurology 2020; 95: 224–225. 32444492 10.1212/WNL.0000000000009850

[33] Aragão MFVV Leal MC Cartaxo Filho OQ , et al. Anosmia in COVID-19 associated with injury to the olfactory bulbs evident on MRI. AJNR Am J Neuroradiol 2020; 41: 1703–1706. 32586960 10.3174/ajnr.A6675 PMC7583088

[34] Bryche B St Albin A Murri S , et al. Massive transient damage of the olfactory epithelium associated with infection of sustentacular cells by SARS-CoV-2 in golden Syrian hamsters. Brain Behav Immun 2020; 89: 579–586. 32629042 10.1016/j.bbi.2020.06.032 PMC7332942

[35] Frasnelli J Schuster B Hummel T. Interactions between olfaction and the trigeminal system: what can be learned from olfactory loss. Cereb Cortex 2007; 17: 2268–2275. 17150985 10.1093/cercor/bhl135

[36] Daiber P Genovese F Schriever VA , et al. Neuropeptide receptors provide a signalling pathway for trigeminal modulation of olfactory transduction. Eur J Neurosci 2013; 37: 572–582. 23205840 10.1111/ejn.12066

[37] Schaefer ML Böttger B Silver WL , et al. Trigeminal collaterals in the nasal epithelium and olfactory bulb: a potential route for direct modulation of olfactory information by trigeminal stimuli. J Comp Neurol 2002; 444: 221–226. 11840476 10.1002/cne.10143

[38] Genovese F Bauersachs HG Gräßer I , et al. Possible role of calcitonin gene‐related peptide in trigeminal modulation of glomerular microcircuits of the rodent olfactory bulb. Eur J Neurosci 2017; 45: 587–600. 27891688 10.1111/ejn.13490

